# Supplementary figures and images for: Upregulation of human GD3 synthase (hST8Sia I) gene expression during serum starvation-induced osteoblastic differentiation of MG-63 cells
Source: PLoS One. 2023 Nov 2;18(11):e0293321. doi: 10.1371/journal.pone.0293321 (PMC10621931; doi:10.1371/journal.pone.0293321)

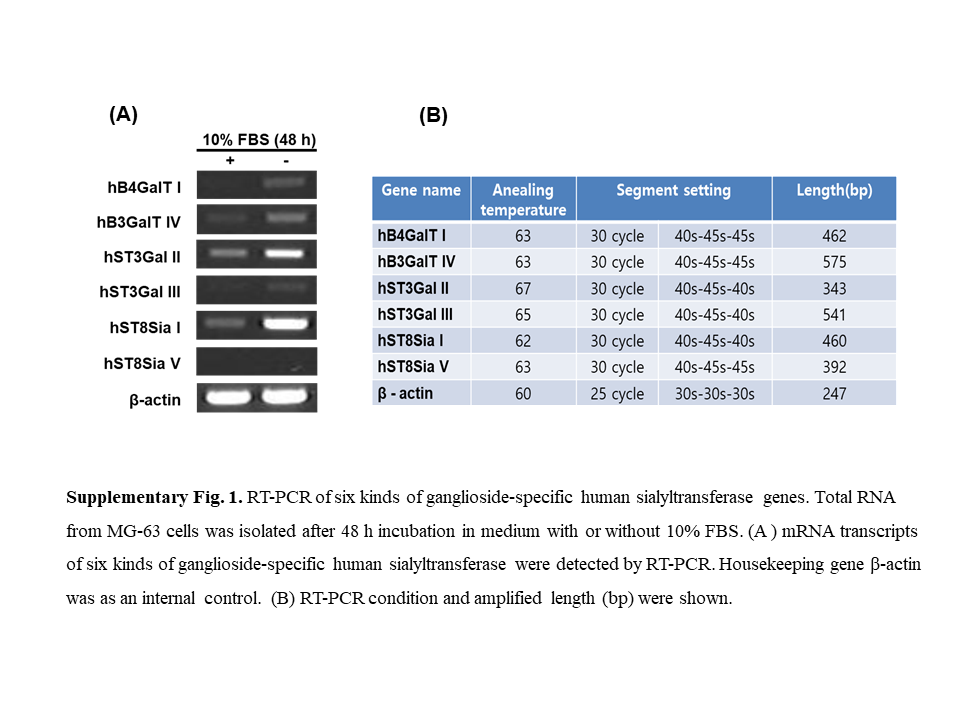

Supplement: S1 Fig — Total RNA from MG-63 cells was isolated after 48 h incubation in medium with or without 10% FBS. (A) mRNA transcripts of six kinds of ganglioside-specific human sialyltransferase were detected by RT-PCR. Housekeeping gene β-actin was used as an internal control. (B) RT-PCR condition and amplified length (bp) were shown. (TIF) [file pone.0293321.s001.tif]

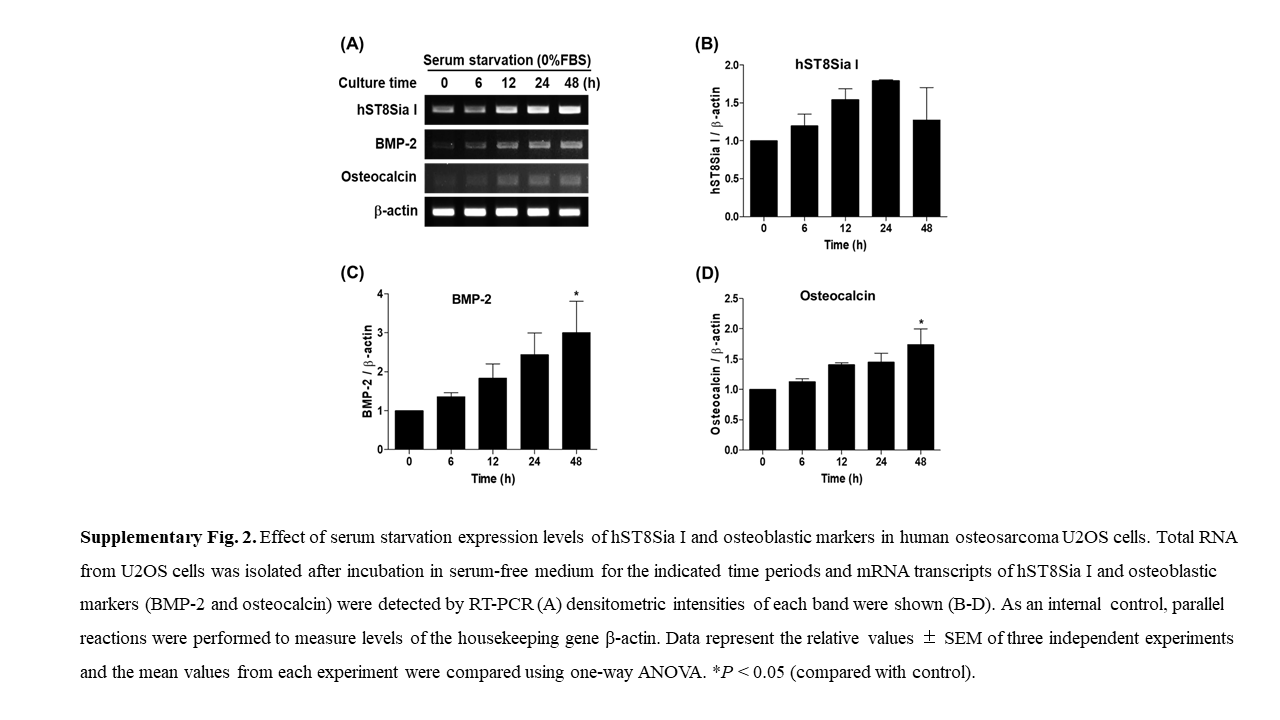

Supplement: S2 Fig — Total RNA from U2OS cells was isolated after 48 h incubation in serum-free medium for the indicated time period and mRNA transcripts of hST8Sia I and osteoblastic markers (BMP-2 and osteocalcin) were detected by RT-PCR (A). Densitometric intensity of each band was shown (B-D). As an internal control, parallel reactions were performed to measure levels of the housekeeping gene β-actin. Data represent the relative values ± SEM of three independent experiments and the mean values from each experiment were compared using one-way ANOVA. * P < 0.06 (compared to control). (TIF) [file pone.0293321.s002.tif]

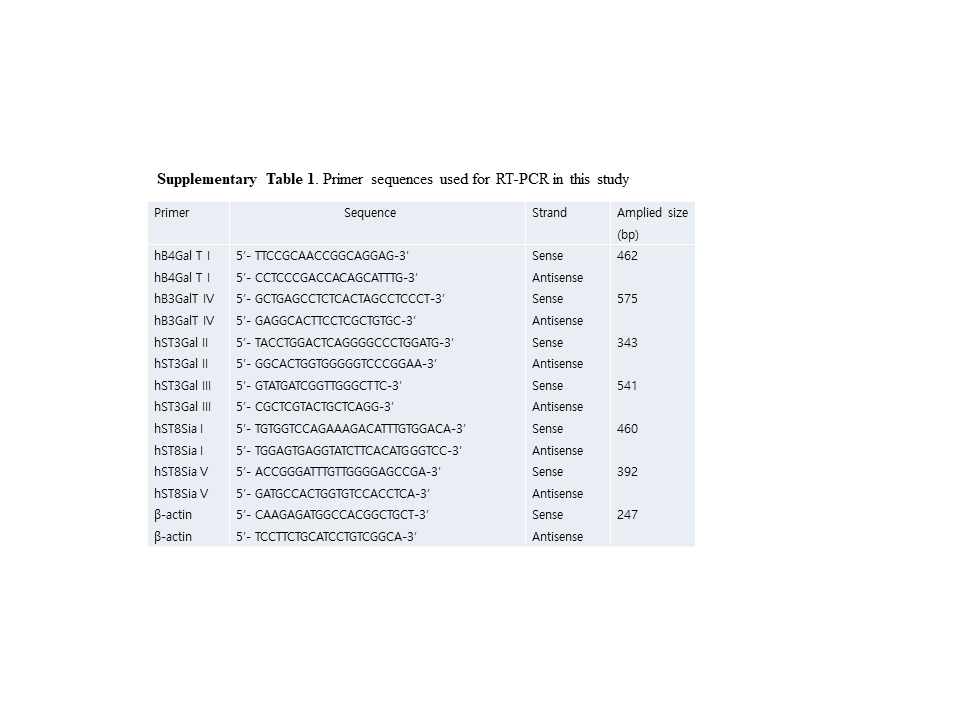

Supplement: S1 Table — (TIF) [file pone.0293321.s004.tif]
