# Supplementary material for: Upregulation of human GD3 synthase (hST8Sia I) gene expression during serum starvation-induced osteoblastic differentiation of MG-63 cells
Source: PLoS One. 2023 Nov 2;18(11):e0293321. doi: 10.1371/journal.pone.0293321 (PMC10621931; doi:10.1371/journal.pone.0293321)
Supplement: S1 File — (PPTX) [file pone.0293321.s003.pptx]

## Slide 1
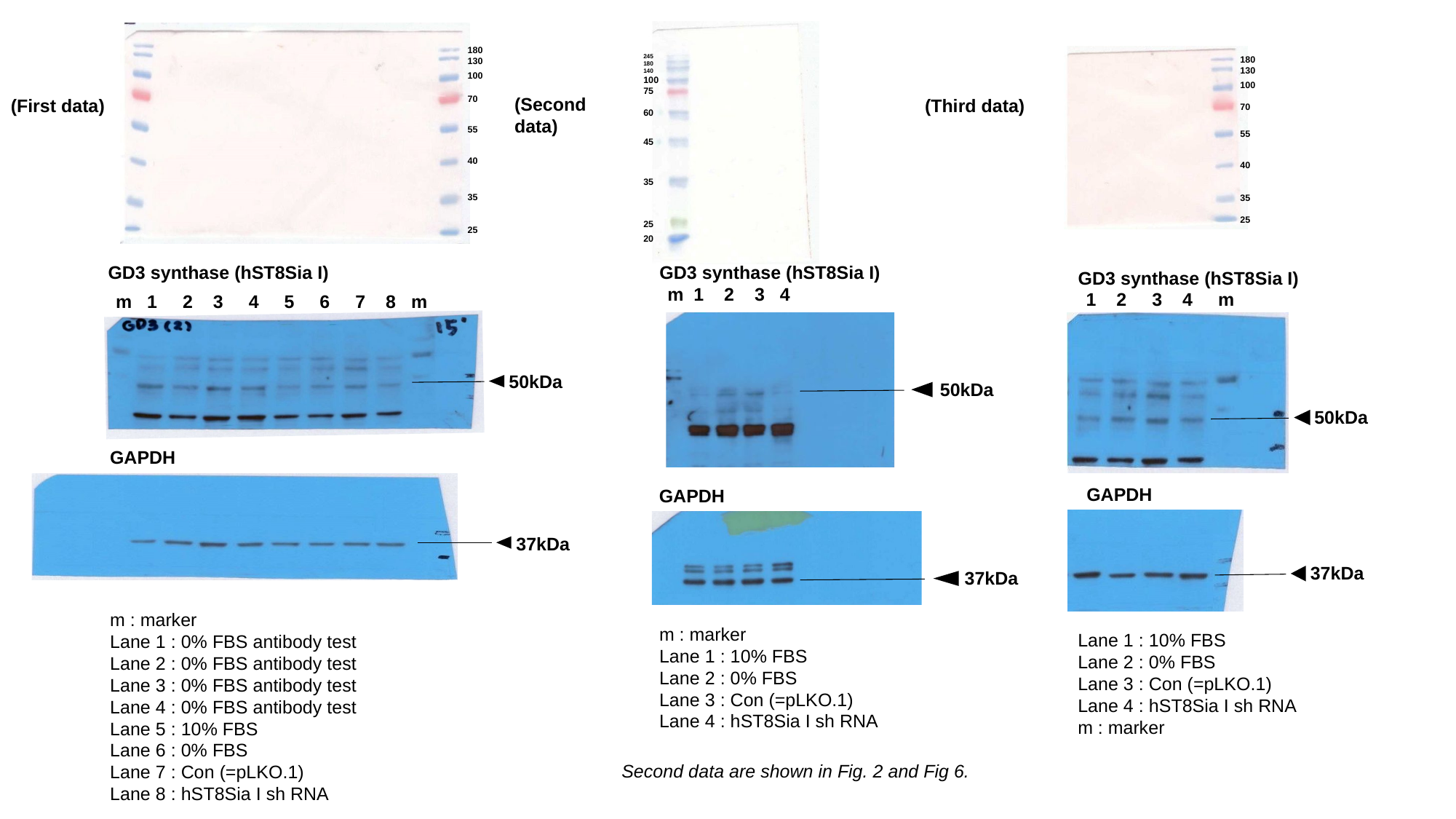

245
180
140
100
75
60
45
35
25
20
GD3 synthase (hST8Sia I)
m 1 2 3 4
 50kDa
GAPDH
 37kDa
m : marker
Lane 1 : 10% FBS
Lane 2 : 0% FBS
Lane 3 : Con (=pLKO.1)
Lane 4 : hST8Sia I sh RNA
180
130
100
70
55
40
35
25
GD3 synthase (hST8Sia I)
m 1 2 3 4 5 6 7 8 m
50kDa
GAPDH
37kDa
m : marker
Lane 1 : 0% FBS antibody test
Lane 2 : 0% FBS antibody test
Lane 3 : 0% FBS antibody test
Lane 4 : 0% FBS antibody test
Lane 5 : 10% FBS
Lane 6 : 0% FBS
Lane 7 : Con (=pLKO.1)
Lane 8 : hST8Sia I sh RNA
180
130
100
70
55
40
35
25
GD3 synthase (hST8Sia I)
1 2 3 4 m
50kDa
GAPDH
37kDa
Lane 1 : 10% FBS
Lane 2 : 0% FBS
Lane 3 : Con (=pLKO.1)
Lane 4 : hST8Sia I sh RNA
m : marker
(Second data)
(First data)
(Third data)
Second data are shown in Fig. 2 and Fig 6.
