# Supplementary material for: Upregulation of human GD3 synthase (hST8Sia I) gene expression during serum starvation-induced osteoblastic differentiation of MG-63 cells
Source: PLoS One. 2023 Nov 2;18(11):e0293321. doi: 10.1371/journal.pone.0293321 (PMC10621931; doi:10.1371/journal.pone.0293321)
Supplement: Attachment — Submitted filename: Replies to reviewer 3 (PONE-D-22-35550).docx [file pone.0293321.s006.docx]

**Answers to Reviewer 3;**

I would like to express my deep appreciation for Reviewer’s comments and agree with all the comments.

**[Reviewer #3]:** The manuscript by An et al Upregulation of Human GD3 Synthase (hST8Sia I) Gene Expression During Serum Starvation-Induced Osteoblastic Differentiation of MG-63 Cells”. In the previously published article, the authors (Yoon et al 2015) demonstrated that hST3Gal V was increased under serum deprived conditions through Runx2 activation by BMP signaling in MG-63 cells. In the current study, the same group observed the upregulation of other human sialyltransferase namely hST8Sia I. Authors first checked the hST8Sia I expression and its regulation BMP2 and osteocalcin in time dependent manner. Increased GD3, luciferase activity, and no effect on osteoblast differentiation with the shRNA experiments. However, authors should address the following comments.

Major comments:
1. In between hST3gal V and hST8Sia I sialyltransferases which one is more significant upregulation upon serum deprivation and which one more important in the osteoblastic differentiation.

**(Answer)** The graph of realtime-PCR results shown in Figure 1 is a graph of the data obtained by performing the experiment three times (expression in the excel file) divided by the value at 0 h. The graph of realtime-PCR in the revised Fig. 1 shows the expression value without dividing the obtained data into 0 h, like the realtime-PCR result of hST3Gal V shown in the previous paper **[*Int. J. Mo. Sci*. 2015, 17(1): E35].** Therefore, as can be seen from the comparison of these two graphs, there was no significant difference in the expression levels by serum deprivation between hST8Sial I and hST3Gal V.

The excel files of realtime-PCR results were provided as supplementary data.

2. Why authors wanted to study only hST8Sia I if all other human sialyltransferases are also upregulated except hST8Sia V. Only the reason that this is highest expression.

**(Answer)** Among the examined human sialyltransferases involved in ganaglioside biosynthesis, as shown in supplementary Figure 1, the expression level of hST8Sia I was the highest, followed by hST3Gal II, but hB4GalT I, hB3GalT IV and hST3Gal III showed low levels. Furthermore, various deletion mutant plasmids of hST8Sia I have been constructed in the previous study.

3. Under same experimental conditions with the same cell line MG 63; authors claiming BMP2, and Osteocalcin are regulating serum starvation in time dependent manner. hST3Gal V is regulating GM3 and regulates osteoblast differentiation. Whereas hST8Sia V is regulating GD3 expression and no effect on osteoblast differentiation. Authors should clear which is most significant regulation for osteoblastic differentiation since both are shown to increase with the SD and BMP2, osteocalcin.

**(Answer)** In the previous study, we demonstrated for the first time that serum deprivation induced G1 arrest of the cell cycle in MG-63 cells and enhanced significantly the transcript levels of marker genes of osteoblast differentiation, such as BMP, osteocalcin and Runx2. At the same time, we also demonstrated that serum deprivation induced not only a significant time-dependent enhancement in both mRNA and protein levels of human GM3 synthase (hST3Gal V), but also the remarkable increase of ganglioside GM3 in MG-63 cells. From these results, we assumed that the increased expressions of hST3Gal V and ganglioside GM3 by serum deprivation induces differentiation of human osteoblastic MG-63 cells.

In this study, we also proved that serum deprivation increased the levels of human GD3 synthase (hST8Sia I) gene and ganglioside GD3 expression as well as the transcript levels of marker genes of osteoblast differentiation, such as BMP and osteocalcin.

From previous and current studies, we have been interested in uncovering the causal relationship between whether differentiation of human osteoblastic MG-63 cells was caused by the increased expressions of hST8Sia I and ganglioside GD3 by serum deprivation, and conversely, whether differentiation of human osteoblastic MG-63 cells by serum deprivation caused the increased expressions of hST8Sia I and ganglioside GD3. To this end, in this study, as described in this paper, we established hST8Sia I-knockdown cells using shRNA and then the changes in the mRNA levels of hST8Sia I and osteoblast differentiation markers, BMP-2, alkaline phosphatase(ALP) and osteocalcin were checked by real-time qRT-PCR analysis. hST8Sia I mRNA expression level was remarkably reduced by 5.7-fold in hST8Sia I knockdown cells compared to control cells (Figure 6A), whereas in hST8Sia I knockdown cells, the mRNA levels of BMP-2, ALP and osteocalcin were 1.6-, 2.4- and 4.8-folds higher than those in control cells, respectively. In addition, protein level of hST8Sia I and ganglioside GD3 expression level were distinctly decreased in hST8Sia I knockdown cells compared to control cells (Figure 6B and 6C). Collectively, these results suggest that expressions of hST8Sia I gene and ganglioside GD3 by serum deprivation have no apparent effect on differentiation of MG-63 cells. In conclusion, we could not clearly identify this causal relationship from the current research results, but it is necessary to investigate the causal relationship between the increased expressions of hST3Gal V and GM3 and induction of MG-63 cell differentiation through establishment of hST3Gal V-knockdown cells using shRNA in the future study.

4. In Fig 1, did authors check the protein levels of BMP-2 and osteocalcin.

**(Answer)** As shown in many papers published previously, we investigated the transcript levels of marker genes of osteoblast differentiation, such as BMP and osteocalcin, but their protein levels were not checked.

5. In supplementary Fig, the expression of hST8Sia I after 48h is seems stronger than the hST3Gal V at the same time (from the results Fig 3 Yoon et al 2015). How do authors justify hST8Sia I had no effect on osteoblastic differentiation.

**(Answer)** As described in the above Q3 answer, we established hST8Sia I-knockdown cells using shRNA and then the changes in the mRNA levels of hST8Sia I and osteoblast differentiation markers, BMP-2, alkaline phosphatase(ALP) and osteocalcin were checked by real-time qRT-PCR analysis. hST8Sia I mRNA expression level was remarkably reduced by 5.7-fold in hST8Sia I knockdown cells compared to control cells (Figure 6A), whereas in hST8Sia I knockdown cells, the mRNA levels of BMP-2, ALP and osteocalcin were 1.6-, 2.4- and 4.8-folds higher than those in control cells, respectively. In addition, protein level of hST8Sia I and ganglioside GD3 expression level were distinctly decreased in hST8Sia I knockdown cells compared to control cells (Figure 6B and 6C). Collectively, these results suggest that expressions of hST8Sia I gene and ganglioside GD3 by serum deprivation have no apparent effect on differentiation of MG-63 cells.

6. How do the authors explain the role of hST8Sia I in osteoblast differentiation. if BMP2 and osteocalcin increasing with SD; SD increases osteoblast differentiation and hST8Sia I had no role in osteoblast differentiation but upregulation in SD.

**(Answer)** As described in the above Q3 answer, we have been interested in uncovering the causal relationship between whether differentiation of human osteoblastic MG-63 cells was caused by the increased expressions of hST8Sia I and ganglioside GD3 by serum deprivation, and conversely, whether differentiation of human osteoblastic MG-63 cells by serum deprivation caused the increased expressions of hST8Sia I and ganglioside GD3. To this end, in this study, as described in this paper, we established hST8Sia I-knockdown cells using shRNA and then the changes in the mRNA levels of hST8Sia I and osteoblast differentiation markers, BMP-2, alkaline phosphatase(ALP) and osteocalcin were checked by real-time qRT-PCR analysis. hST8Sia I mRNA expression level was remarkably reduced by 5.7-fold in hST8Sia I knockdown cells compared to control cells (Figure 6A), whereas in hST8Sia I knockdown cells, the mRNA levels of BMP-2, ALP and osteocalcin were 1.6-, 2.4- and 4.8-folds higher than those in control cells, respectively. In addition, protein level of hST8Sia I and ganglioside GD3 expression level were distinctly decreased in hST8Sia I knockdown cells compared to control cells (Figure 6B and 6C). Collectively, these results suggest that expressions of hST8Sia I gene and ganglioside GD3 by serum deprivation have no apparent effect on differentiation of MG-63 cells. In conclusion, we could not clearly identify this causal relationship from the current research results, but it is necessary to investigate the causal relationship between the increased expressions of hST3Gal V and GM3 and induction of MG-63 cell differentiation through establishment of hST3Gal V-knockdown cells using shRNA in the future study.

7. Authors should reframe the title. Which covers “hST8Sia I no effect on osteoblast differentiation” instead of current one which is misleading.

(Answer) As described in this study, our study focuses on investigating transcriptional regulatory mechanism of hST8Sia I gene by serum deprivation. In this study, we firstly found that the expression levels of human GD3 synthase (hST8Sia I) gene and ganglioside GD3 were increased by serum deprivation in MG-63 cells. At the same time, the transcript levels of marker genes of osteoblast differentiation, such as BMP and osteocalcin were increased by serum deprivation in MG-63 cells, indicating differentiation of human osteoblastic MG-63 cells. On the basis of these findings, to elucidate the molecular mechanism of upregulation of hST8Sia I gene expression induced by serum deprivation, the promoter region to mediate transcriptional upregulation of hST8Sia I gene in serum-deprived cells was functionally characterized, and we also showed involvement of p38MAPK/NF-κB signaling pathway in hST8Sia I transcriptional activation induced by serum deprivation.

8. Schematic representation should be included.

(Answer) According to the reviewer’s suggestion, schematic representation has been shown in Fig. 7.
