# Supplementary material for: Upregulation of human GD3 synthase (hST8Sia I) gene expression during serum starvation-induced osteoblastic differentiation of MG-63 cells
Source: PLoS One. 2023 Nov 2;18(11):e0293321. doi: 10.1371/journal.pone.0293321 (PMC10621931; doi:10.1371/journal.pone.0293321)
Supplement: Attachment — Submitted filename: Replies to reviewer (PONE-D-23-19970).docx [file pone.0293321.s007.docx]

**Answers to Reviewer;**

Thank you for submitting your manuscript to PLOS ONE. After careful consideration, we feel that it has merit but does not fully meet PLOS ONE’s publication criteria as it currently stands. Therefore, we invite you to submit a revised version of the manuscript that addresses the points raised during the review process.

It is not clear how well serum starvation reproduces the impact of nutrient starvation on the organism. The introduction seems too speculative in this regard and does not contribute to the understanding of the experimental design. Serum starvation is a common maneuver to induce a differentiation response in many cell types and there is no need to force a correlation with undernutrition. Moreover, it is difficult to determine whether the impact of serum starvation is due to a nutritional deficiency, oncotic pressure, or growth fact withdrawal without the addition of protein (albumin) in the same quantity provided by serum.

**(Answer)** According to the reviewer’s suggestion, we revised the introduction with a focus on experimental design rather than too speculative (page 2, Introduction section: lines 2-19 were deleted in the revised MS).

Although NFkB mutation has the largest effect in promoter activity it does not seem to discriminate between 0% and 10% of FBS, suggesting it is not the determining factor in starvation-induced differential expression of hST8Sia. In contrast, the mutation in c-Ets site does not modify basal (10% FBS) expression while abrogating serum starvation-induced expression.  Thus, the sentence "suggesting that NF-κB is implicated in serum starvation-induced transcriptional activity in MG-63 cells" is not supported by the data and should be modified to reflect the role of NF-kB as a major driver of hST8Sia expression and c-Ets as driving the selective higher gene expression in serum-starved cells. The role of NF-kB seems to be the opposite of what was reported for other cell types, however, no possible explanation is provided for it in Discussion.

**(Answer)** As described in the MS, although mutations in AP-1, CREB, and c-Ets-1 binding sites showed a significant decrease in luciferase activity upon serum starvation compared to wild-type pGL3-1146/-646, mutation of the NF-κB binding site almost completely eliminated the increased luciferase activity, both in 0% and 10% FBS-containing media. Along with the results of NF-κB-specific inhibitor (PDTC) treatment and *in vivo* ChIP assay, this result indicates that the binding of NF-κB to its recognition site is essential for transcriptional activation of *hST8Sia I* in MG-63 cells.

As the reviewer mentioned, the sentence "suggesting that NF-κB is implicated in serum starvation-induced transcriptional activity in MG-63 cells" has been amended to the sentence “indicating that the binding of NF-κB to its recognition site is essential for transcriptional activation of *hST8Sia I* in MG-63 cells” in the revised MS (page 10, lines 6-8: red sentence).

As the reviewer also mentioned, the role of NF-kB as opposed to that reported for other cell types has been described in the Discussion section of revised MS (page 13, lines

17-21: red sentences)

The crosstalk between NF-kB and p38 signaling pathways in hST8Sia expression could be easily assessed by any of the different assays (gene reporter, ChIP) used in the manuscript. It seems like a missed opportunity, but most importantly figure 7 is speculative without the direct proof that p38 is actually modulating NF-kB activity.

**(Answer)** As the reviewer can see in many papers published previously, signaling pathways involved in transcriptional regulation of human sialyltransferases have been investigated by promoter assays using specific inhibitors of kinases associated with signaling pathways.

We agree with the reviewer’ suggestion that the crosstalk between NF-kB and p38 signaling pathways in hST8Sia I expression needs to be assessed by any of the different assays (gene reporter, ChIP). We will conduct this experiment in similar studies in the future.

According to the reviewer’s suggestion, Fig. 7 has been deleted in the revised MS.

The sentences "Collectively, these results suggest that expression of hST8Sia I and ganglioside GD3 have no apparent effect on the differentiation of MG-63 cells" (page 13) and "Suppression of GD3 expression by knockdown of hST8Sia I did not cause any reduction in gene expression levels of BMP, ALP, and osteocalcin known as osteoblast differentiation markers" does not represent the potential role of hST8Sia I and GD3 in differentiation. The data in Figure 6 point to a negative effect on differentiation markers in particular on osteocalcin expression. Any other effect of serum starvation such as G1 arrest is impacted by the knockdown of hST8Sia? Knockdown of hST8Sia has any impact on the expression of other sialyltransferases?

**(Answer)** Since the differentiation of mammalian cells is known to be preceded by G1 arrest of cell cycle, in the previous study, we have also examined whether serum deprivation induces G1 arrest of cell cycle in MG-63 cells. After MG-63 cells were incubated under serum-free condition for various times, cells were collected and the cell cycle profile was analyzed by flow cytometry. The result showed that the percentage of cells in G1 phase was increased time-dependently by serum deprivation, whereas the percentages of S and G2 phases were decreased. Cells in sub-G1 phase were not observed, indicating that serum deprivation did not cause cell death. In addition, morphology of MG-63 cells became branched and elongated time-dependently [*Int. J. Mo. Sci*. 2015, 17(1): E35].

From previous and present studies, we have been interested in uncovering the causal relationship between whether differentiation of human osteoblastic MG-63 cells was caused by the increased expressions of hST8Sia I and ganglioside GD3 by serum deprivation, and conversely, whether differentiation of human osteoblastic MG-63 cells by serum deprivation caused the increased expressions of hST8Sia I and ganglioside GD3. To this end, in this study, as described in this paper, we established hST8Sia I-knockdown cells using shRNA and then the changes in the mRNA levels of hST8Sia I and osteoblast differentiation markers, BMP-2, alkaline phosphatase(ALP) and osteocalcin were checked by real-time qRT-PCR analysis. hST8Sia I mRNA expression level was remarkably reduced by 5.7-fold in hST8Sia I knockdown cells compared to control cells (Figure 6A), whereas in hST8Sia I knockdown cells, the mRNA levels of BMP-2, ALP and osteocalcin were 1.6-, 2.4- and 4.8-folds higher than those in control cells, respectively. In addition, protein level of hST8Sia I and ganglioside GD3 expression level were distinctly decreased in hST8Sia I knockdown cells compared to control cells (Figure 6B and 6C). Collectively, these results suggest that expressions of hST8Sia I gene and ganglioside GD3 by serum deprivation have no apparent effect on differentiation of MG-63 cells. In conclusion, we could not clearly identify this causal relationship from the present study. However, the expression levels of ganglioside GM3 synthase (hST3Gal V) and its product GM3 have been also markedly increased during MG-63 cell differentiation by serum starvation ([*Int. J. Mo. Sci*. 2015, 17(1): E35], it is necessary to investigate the causal relationship between the increased expressions of hST3Gal V and GM3 and induction of MG-63 cell differentiation through establishment of hST3Gal V-knockdown cells using shRNA in the future study.
